# Characterization of the Temporal Dynamics of the Endothelial–Mesenchymal-like Transition Induced by Soluble Factors from Dengue Virus Infection in Microvascular Endothelial Cells

**DOI:** 10.3390/ijms26052139

**Published:** 2025-02-27

**Authors:** Jenny Paola Alfaro-García, Carlos Alberto Orozco-Castaño, Julián Andrés Sánchez-Rendón, Herley Fernando Casanova-Yépes, Miguel Vicente-Manzanares, Juan Carlos Gallego-Gómez

**Affiliations:** 1 Grupo Medicina de Translación—Facultad de Medicina, Universidad de Antioquia, Medellín 050010, Colombia; jenny.alfaro@udea.edu.co; 2 Grupo de Biología del Cáncer, Instituto Nacional de Cancerología, Bogotá 111511, Colombia; corozco@cancer.gov.co; 3 Grupo de Coloides—Facultad de Ciencias Exactas y Naturales, Universidad de Antioquia, Medellín 050010, Colombia; buzon4@gmail.com (J.A.S.-R.); herley.casanova@udea.edu.co (H.F.C.-Y.); 4 Molecular Mechanisms Program, Centro de Investigación del Cáncer, Instituto de Biología Molecular y Celular del Cáncer, Consejo Superior de Investigaciones Científicas (CSIC)—University of Salamanca, 37007 Salamanca, Spain

**Keywords:** endothelial permeability, plasticity, trans-differentiation, conditioned culture medium, dengue virus

## Abstract

Dengue virus (DV) infection poses a severe life-threatening risk in certain cases. This is mainly due to endothelial dysregulation, which causes plasma leakage and hemorrhage. However, the etiology of DV-induced endothelial dysregulation remains incompletely understood. To identify the potential mechanisms of endothelial dysregulation caused by DV, the effects of conditioned media from Dengue virus (CMDV) on the mechanics and transcriptional profile of the endothelial cells were examined using permeability assays, atomic force microscopy, In-Cell Western blot and in silico transcriptomics. Exposure of HMEC-1 cells to the CMDV increased endothelial permeability and cellular stiffness. It also induced the expression of the key proteins associated with endothelial-to-mesenchymal transition (EndMT). These data support the notion that the DV promotes endothelial dysfunction by triggering transcriptional programs that compromise the endothelial barrier function. Understanding the molecular mechanisms underlying DV-induced endothelial dysregulation is crucial for developing targeted therapeutic strategies to mitigate the severe outcomes associated with dengue infection.

## 1. Introduction

Dengue virus (DV) is a flavivirus transmitted by *Aedes* mosquitoes [1]. The emergence of dengue as a global threat is attributed to human expansion into rural areas and climate change. Accordingly, a substantial portion of the world’s population will be eventually exposed to this disease [2,3]. Efforts to develop a universal vaccine against DV have faced challenges due to the intrinsic nature of the virus and the existence of several different serotypes [4,5]. Dengue infection is often asymptomatic, with only selected cases progressing to severe forms of the disease. However, the critical issue in public health lies in the absence of effective treatments for severe cases [6,7].

This research addresses the emergence of endothelial dysfunction as a complication associated with severe dengue. Endothelial dysfunction often results in vascular leakage, hemorrhage, shock, and organ failure [8,9]. Experimentally, endothelial dysfunction is studied by measuring alterations in the permeability of the endothelial monolayers, which is driven by the interplay between the viral determinants and host proteins [5,10]. While endothelial dysfunction has been linked to cytokine storms, immune-based therapies have demonstrated the limited effectiveness in preventing or mitigating this condition, likely due to its onset during the post-febrile phase [11,12]. This suggests that the DV may alter endothelial cell function in an immune-independent manner. In addition to the direct effects on immune cells and inflammation, cytokine release during a cytokine storm (including IL-10, TNF-α, GM-CSF, IFN-γ, NF-κβ, IL-15, IL-8, CCL2, IL-6, CCL4, and others [8]) may have direct effects on endothelial cells. One of such effects is the endothelial-mesenchymal transition (EndMT). EndMT represents a reversible trans-differentiation event associated with physiological processes like development or wound healing, and pathological conditions such as fibrosis and cancer [13]. Furthermore, this phenomenon has also been linked to septic shock induced by bacteria, and hypoxia [13,14,15,16,17]. Notably, other viruses induce epithelial-mesenchymal transitions (EMT), which result in a similar cellular outcome, that is, trans-differentiation of barrier-forming cells into more motile and loosely connected fibroblast-like cells. EMT and EndMT have common underlying molecular principles, including shared signaling pathways and mediators [18]. Two key examples of EMT-inducing viruses are the sarcoma-associated herpesvirus and SARS-CoV-2 [19,20].

Our previous findings have revealed that the conditioned media from dengue virus (CMDV) modify the expression and function of the host proteins involved in cell migration [21]. That study reported significant changes in the migratory capability and cellular phenotype in microvascular endothelial cells exposed to conditioned media, which contained cytokines like IL-3, TNF-α, FGF, CCL5, and others [21]. Additionally, DV induces morphological changes in epithelial cells consistent with increased motility and matrix remodeling abilities, such as increased stress fibers and membrane protrusions [22,23]. Based on these data, we posit that the DV triggers EndMT in response to soluble factors (that can be of cellular and/or viral origin) generated during infection. Successfully addressing this hypothesis would provide an explanation for the limited efficacy of immunological treatments and the absence of significant post-mortem evidence of substantial tissue damage in this context [24,25,26].

In this study, in vitro experiments were performed to evaluate the impact of the soluble factors produced during the DV infection on cellular stiffness (which is a marker of EMT/EndMT [27]) and the expression of EndMT protein markers. Furthermore, we assessed the potential of imatinib, a multitarget receptor tyrosine kinase inhibitor, to reverse endothelial trans-differentiation and dysfunction [28]. Moreover, this study describes an in silico analysis that assesses the effects of selected DV-dependent factors on the endothelial transcriptome.

## 2. Results

### 2.1. CMDVs Increase Endothelial Permeability

To investigate the effect of the conditioned media from dengue virus (CMDV) on endothelial cell permeability, HMEC-1 endothelial cells were cultured to confluency. The cells were then incubated with the CMDV, followed where indicated by treatment with the tyrosine kinase pan-blocker imatinib mesylate (treated groups) or DMSO (vehicle) as described in Section 4 (Material and Methods). Controls included untreated cells (control group), and cells treated with TGF-β1 (positive control). Trans-endothelial electrical resistance (TEER) measurements were performed at 48 h and 120 h. After 120 h of incubation with the CMDV, the indicated groups were treated with imatinib and the TEER was measured at 48 h and 120 h post-imatinib, that is, 168 h and 240 h after the beginning of the experiment.

**Figure 1:**
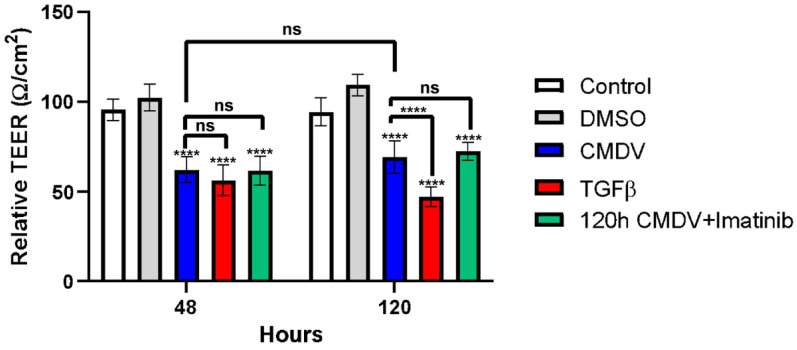
**CMDV increases endothelial permeability.** Relative trans-endothelial electrical resistance (TEER) of HMEC-1 cells treated with the conditioned media from dengue virus (CMDV), TGF-β, or the CMDV + imatinib was measured at 48 and 120 h. Significant reductions in the TEER were observed in the CMDV, TGF-β, and the CMDV + imatinib-treated groups compared to control cells at both time points. At 120 h, TGF-β treatment further decreased the TEER compared to the CMDV and CMDV + imatinib treatments. No significant changes (ns) were observed between the CMDV, TGF-β, and CMDV + imatinib at 48 h. The Mann–Whitney U test was employed for statistical analysis, and *p*-values less than 0.05 were considered statistically significant. **** *p* < 0.0001.

As shown in Figure 1, a significant reduction in TEER was observed in cells treated with CMDV and TGF-β compared to the control group at both 48 and 120 h. At 120 h, TGF-β treatment resulted in a significant decrease in TEER compared to the CMDV, suggesting that the CMDV compromises the integrity of the endothelial barrier in a sustained manner. The addition of imatinib to the CMDV-treated cells had no significant effect on TEER, suggesting that the mechanism by which the CMDV compromises endothelial barrier function may not depend on c-ABL, the main target of the compound.

### 2.2. CMDVs Modulate the Expression of the Proteins Associated with EndMT

**Figure 2:**
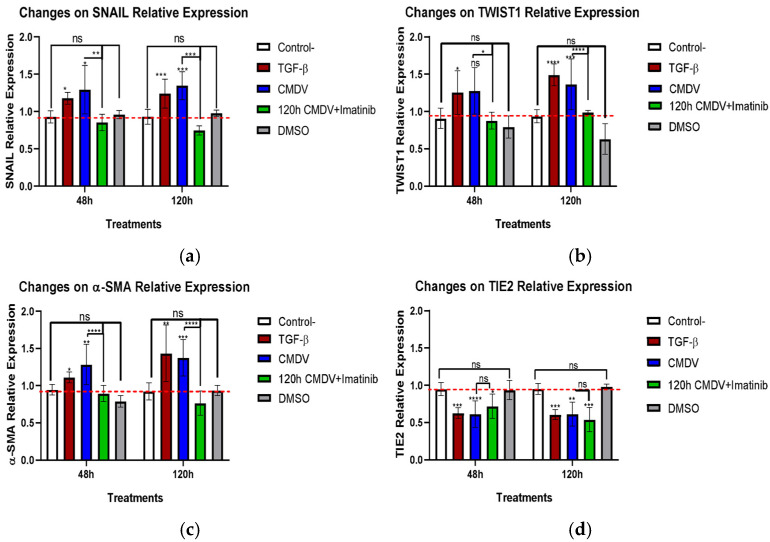
**Effects of CMDV and imatinib on the expression of EndMT-associated proteins in HMEC-1 cells.** Relative expression levels of (**a**) SNAIL, (**b**) TWIST1, (**c**) α-SMA, and (**d**) TIE2 were measured in HMEC-1 cells treated with the conditioned media from dengue virus (CMDV), TGF-β, or imatinib, as well as control treatments (DMSO and untreated cells) at 48 and 120 h. CMDV and TGF-β treatments significantly upregulated the expression of EndMT-associated proteins (SNAIL, TWIST1, α-SMA) and downregulated TIE2 expression compared to the controls. Imatinib treatment partially reduced the expression of these EndMT markers but did not counter the effect of CMDV on TIE2 levels. Dashed red line denotes the baseline of control cells and is shown for reference purposes only. Statistical significance is indicated as follows: * *p* < 0.05, ** *p* < 0.01, *** *p* < 0.001, **** *p* < 0.0001, ns = not significant. Data are presented as mean ± SEM from three independent experiments.

To determine if the CMDV modulate the relative expression of the proteins associated with EndMT trans-differentiation, HMEC-1 cells were treated with the CMDV, and the expression of specific proteins was measured by ICW. These experiments revealed moderate, yet consistent, changes in the expression of selected proteins, as depicted in Figure 2a–c.

The CMDV increased the expression of SNAIL, TWIST1, and α-SMA compared to untreated HMEC-1 cells (Figure 2a–c). Conversely, we observed a marked reduction in the protein levels of the endothelial receptor TIE2 (Figure 2d).

Interestingly, the addition of imatinib to HMEC-1 cells exposed to the CMDV decreased the expression of SNAIL, TWIST1 and α-SMA to basal levels. This suggests that imatinib may partially inhibit the trans-differentiation process induced by the CMDV. Notably, imatinib did not counter the inhibitory effect of the CMDV in the TIE2 expression, supporting that the inhibitory effect of imatinib on EndMT is limited and gene-dependent.

### 2.3. The CMDV Increase Endothelial Cell Stiffness

**Figure 3:**
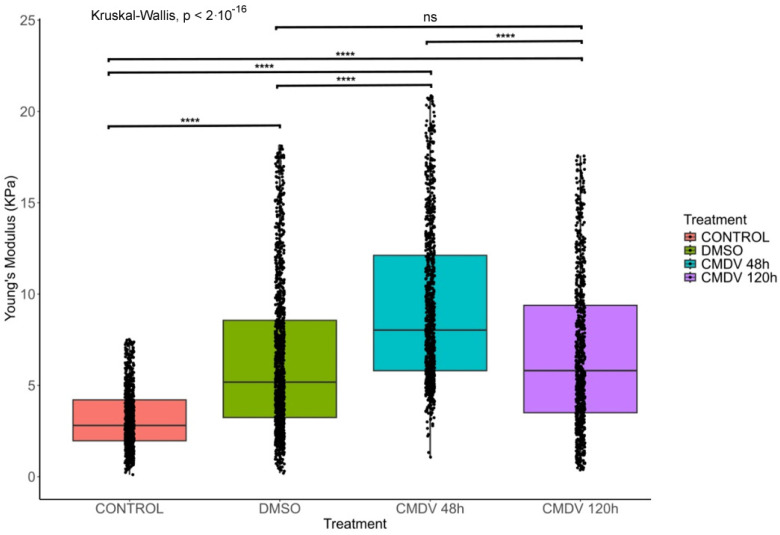
**CMDV induces a temporal increase in cellular stiffness in HMEC-1 cells.** Box plot showing Young’s modulus (which correlates with cell stiffness) of HMEC-1 cells under different treatments: control (untreated), DMSO, CMDV (48 and 120 h). CMDV treatment significantly increased cell stiffness after 48 h, which is the highest stiffness observed. In contrast, the stiffness decreased after 120 h. Statistical significance was determined using the Kruskal–Wallis test, with *p*-values indicating significant differences between the groups. Data are presented as mean ± SEM. ns, not significant; **** *p* < 0.0001.

To determine the impact of the CMDV on cell stiffness, we performed liquid force spectroscopy experiments. These revealed that the CMDV increases cell stiffness in endothelial microvascular cells (Figure 3). These alterations are temporal and heterogeneous, with a peak at 48 h that declines after 120 h, although the values are still more elevated than those seen in control cells.

### 2.4. DV-Induced Soluble Factors Promote Expression of Host Proteins Involved in EndMT

**Figure 4:**
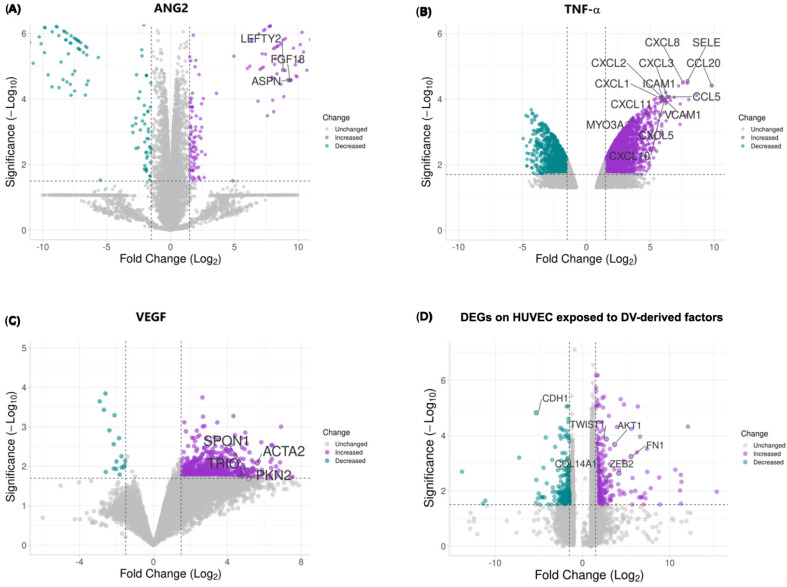
**Differentially expressed genes in HUVEC cells exposed to ANG2, TNF-α, VEGF, and the combined factors.** Volcano plots display the transcriptional changes in Human Umbilical Vein Endothelial Cells (HUVECs) exposed to individual and combined dengue virus-derived soluble factors. (**A**) Effect of ANG2 exposure on gene expression. (**B**) Effect of TNF-α exposure on gene expression. (**C**) Effect of VEGF exposure on gene expression. (**D**) Effect of the combined exposure to ANG2, TNF-α, and VEGF. The x-axis represents the log_2_ fold change, with positive values indicating upregulation and negative values indicating downregulation of genes. The y-axis represents the statistical significance (−log_10_ *p*-value). Genes with increased expression are shown in purple, while those with decreased expression are in teal. Key differentially expressed genes associated with endothelial permeability, cellular stiffness, and endothelial-to-mesenchymal transition (EndMT) are labeled. The volcano plots are scaled consistently to enable direct visual comparisons across conditions.

**Figure 5:**
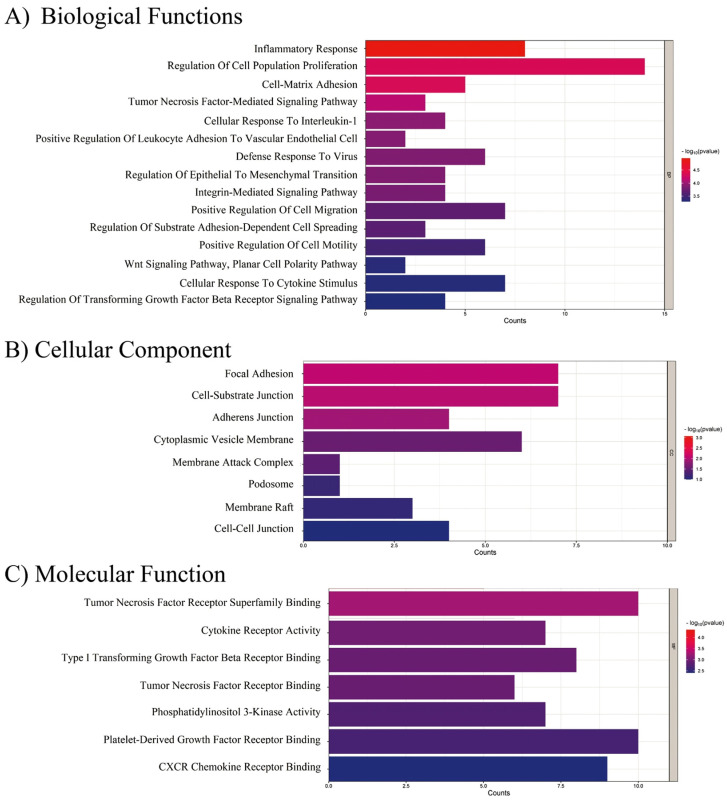
**Gene ontology (GO) enrichment analysis of DEGs in HUVEC cells exposed to dengue virus-derived factors.** The GO enrichment analysis highlights the significantly enriched biological processes, cellular components, and molecular functions associated with differentially expressed genes (DEGs) in HUVEC cells exposed to ANG2, TNF-α, and VEGF. (**A**) Biological Functions: Enriched processes include the inflammatory response, regulation of cell population proliferation, cell-matrix adhesion, and epithelial-to-mesenchymal transition (EMT). (**B**) Cellular Components: Enriched components include focal adhesion, cell-substrate junctions, adherens junctions, and membrane raft. (**C**) Molecular Function: Significant functions include tumor necrosis factor receptor superfamily binding, cytokine receptor activity, and phosphatidylinositol 3-kinase activity. The color gradient represents the significance level (−Log_10_ *p*-value), and the length of the bars indicates the count of genes associated with each GO term.

To explore the impact of DV-induced soluble molecules on an endothelial cell transcriptomic profile, we performed a meta-analysis integrating data from diverse studies using expression arrays of Human Umbilical Vein Endothelial Cells (HUVEC) exposed individually to soluble factors induced by the DV infection, including ANG2, TNF-α, and VEGF. Specific study details and experimental conditions are summarized in Table 1. Differential expression analysis results are depicted in a volcano plot (Figure 4), illustrating the gene expression profile of HUVEC cells exposed to these soluble factors compared to untreated controls, specific information on the DEGs induced by individual stimuli (ANG2, TNF-α, and VEGF) as well as by the combined approach can be found in Supplementary Table S1. Exposure led to the upregulation of mesenchymal-associated genes (e.g., ZEB2, TWIST1, FN) and a decrease in E-Cadherin (CDH1) levels. Then selected the top 100 differentially expressed genes (DEGs) for the enrichment analysis, revealing significant involvement in biological processes such as the Epithelial-to-Mesenchymal Transition (EMT), positive regulation of cell migration, substrate adhesion regulation, adhesion-dependent cell spreading, positive cell motility, and TGF-β receptor signaling regulation (Figure 5A).

Furthermore, our analysis unveiled specifically enriched cellular components (focal adhesion, adherens junctions, podosomes) and molecular functions related to these processes (Figure 5B,C) that provide a molecular basis for the observed trans-differentiation in endothelial cells induced by DV-derived soluble factors.

Finally, we performed an exploratory gene-drug interaction analysis using The Drug Gene Interaction Database (DGIdb, RRID:SCR_006608). For this analysis, we employed the top 100 DEGs identified in the differential expression analysis. Our findings indicate that the differentially expressed genes resulting from the exposure of HUVEC cells to DV-soluble factors could be targeted by several FDA-approved drugs (Table 2). This highlights potential repurposing opportunities for existing medications to address the molecular consequences of DV-induced endothelial changes.

### 2.5. EndMT-Associated Transcriptional Changes in Response to CMDV and Individual Stimuli

To assess the transcriptional response of key EndMT-associated genes, we compared their expression following stimulation with individual factors (ANG2, TNF-α, and VEGF) and their combined effect, emulating the conditioned media of Dengue virus (Supplementary Tables S2 and S3). CMDVs represent the synergistic action of these three factors and consequently induced a marked upregulation of TWIST1, ZEB2, FN1, and COL1A1, which are canonical markers of EndMT. Notably, ANG2 independently upregulated ZEB2, FN1, and TGF-β1, while TNF-α alone induced significant changes in SNAI2, TWIST2, and COL1A1. In contrast, VEGF alone upregulated TWIST1, ACTA2, and NOTCH1. Interestingly, CDH1 (E-cadherin, CD324), which is classic epithelial marker, was not significantly downregulated (Supplementary Tables S2 and S3). These findings suggest that the combination of ANG2, TNF-α, and VEGF exerts a stronger and more coordinated effect on EndMT than individual stimuli, reinforcing the notion that the CMDV is a potent inducer of mesenchymal transition in endothelial cells through the induction of multiple mediators.

## 3. Discussion

Endothelial dysfunction associated with severe dengue is a synergistic response to multiple factors that involve the pathogen and the host [10]. For the present work, the CMDV were obtained as described in Alvarez et al. [21] and Escudero et al. [29]. This approach allowed us to evaluate the effects of the soluble factors produced in response to DV infection and not driven by the virion itself, or the infection process (Supplementary Figure S1a,b). This is important because the deleterious effects associated with endothelial dysfunction in severe dengue patients appear in the post-febrile phase, when viral titers are stable and trending lower than in the febrile phase [11,12]. This suggests that endothelial dysfunction is a delayed cellular response due to exposure to host- or viral-produced factors.

CMDV increases cellular permeability, which agrees with the hypothesis that the DV infection induces soluble factors (endogenous or virus-produced) that cause endothelial dysfunction [21,29]. These effects could be mediated by a decrease of the levels of proteins involved in the maintenance of the endothelial phenotype, an increase in the levels of proteins that mediate mesenchymal behavior and/or post-translational modifications that modulate the function of receptors such as TIE2 [30]. The effect is clearest at 120 h. However, the effect of the CDMV is significantly different from that of TGF-β1, which increases in a time-dependent effect. On the other hand, 120h exposure to the CMDV and later to the Tyr kinase inhibitor imatinib do not restore endothelial integrity, suggesting that the endothelial dysfunction induced by the CMDV is relatively insensitive to the targets of imatinib, e.g., c-ABL. In this regard, imatinib blocks the Slug (Snail2) expression in cancer cells [31], suggesting that the CMDV may induce EndMT by multiple pathways, some of which may be imatinib-sensitive while others are imatinib-insensitive. 

DV infection increases cellular permeability [32,33,34,35], and its severity is related to the strain pathogenicity [10,36]. PBMCs, inflammatory cytokines, e.g. TNF-α, or overexpressed ANG2 [23,36,37,38], as well as reduced levels of PECAM-1 and VE-CAD, and upregulation of sVCAM-1 and E-selectin (CD62E) [39,40,41,42] may also contribute to the loss of endothelial integrity [43,44,45]. In addition to host-produced soluble factors, viral NS1 significantly contributes to endothelial dysfunction. NS1 increases endothelial permeability [29,34,44,46,47,48]. Several mechanisms have been proposed, including the destabilization of intercellular junctions and the cellular glycocalyx by activation of cathepsin/heparinase and sialidases [10,49,50,51]. NS1 also induces proinflammatory factors (cytokines like IL-6 and chemokines such as IL-8, MCP1, RANTES, and others) and growth factors such as TGF-α, FGF-2, GM-CSF and others that may potentiate the inflammatory response. Some of these factors may induce or contribute to the onset of EndMT. Finally, some of these factors promote cell motility, which may contribute to endothelial destabilization in severe cases [21,52,53]. 

Increased cellular permeability is not a marker of EndMT per se. However, elevated expressions of SNAIL and TWIST1, and downregulated TIE2 [13] are consistent with the onset of EndMT. Specifically, the CMDV slightly increased the expression of SNAIL, TWIST1, and α-SMA at 48 and 120 hours, similar to TGF-β1, a potent inducer of EndMT [54]. These data are consistent with a previous report indicating that the CMDV triggers the expression of N-cadherin and vimentin, and downregulates VE-cadherin and ZO-1, which is consistent with an EndMT-like transition [29]. TWIST and SNAIL are likely mediators of the CMDV-induced EndMT-like phenomena described here, since it has been shown that TWIST induces EndMT in a lung fibrosis model [55] and TWIST-dependent EndMT under shear stress is modulated by dexamethasone [56]. The effect of TGF-β seems modest, due to the low dose used to induce mesenchymal transition [54,57,58,59,60], which is in itself a heterogenous event [14,59,61,62,63,64]. 

Despite these variations, the results were consistent across treatments, including TIE2 downregulation. Imatinib reduced the expression of mesenchymal proteins, even when administered late. However, TIE2 did not return to basal levels. c-ABL inhibition by imatinib and TIE2 downregulation may be linked through the role of TIE2 in the angiotensin pathway [65]. It is feasible that early use of imatinib could mitigate endothelial dysfunction, reducing plasma leakage in severe dengue. In this regard, imatinib alleviates fibrotic diseases, which bear an essential EndMT component [66,67], such as fibrotic lung disease [68] and systemic sclerosis [69].

In addition to its effect on cellular transitions, the expression of SNAIL during RNA virus infections such as DV [70] activates the RLR pathway and reduces viral replication [71]. Additionally, Kaposi’s sarcoma virus [19] and SARS-CoV2 [20] can induce EMT [72]. Moreover, while the expression of TWIST1 has not been described in the context of DV infection, TWIST1 is a central transcriptional regulator of EMT and EndMT, together with SNAIL, SLUG, and ZEB1 [13]. This reinforces the notion that the DV may induce EndMT in this context [29,73,74]. These results were further supported by in silico data aimed to simulate the effect of the conditioned medium in endothelial cells (including VEGF because of its increase during DV infection [43]). Our in silico approach identifies a number of potential candidates, but its implication in DENV-induced trans-differentiation processes remains to be validated experimentally. These data indicate that ZEB2, TWIST1, FN are upregulated by DV infection, whereas E-CAD/CD324 is downregulated [13]. Globally, the DEGs observed in our in silico approach are associated with positive regulation of cell migration and motility, substrate adhesion and spreading, and TGF-beta receptor signaling, supporting the experimental results included in the first part of this study and providing a suitable molecular explanation for the increased stiffness caused by the CMDV. Some of these DEGs were also detected in a previous study which also used CMDV-treated HMEC-1 cells [21].

The in silico approach could identify not only possible therapeutic targets, but also regulators of the former, independent of their appearance in the DEG list, e.g., c-ABL, which will be the focus of future research. In this regard, imatinib counteracts EndMT, the DV viral cycle and endothelial permeability and cellular migration, likely through its effect on the cytoskeleton [13,29,75,76,77,78,79,80]. According to this, increased α-SMA expression induced by the CMDV was curbed by imatinib. α-SMA is involved in actin and stress fibers reorganization, cellular rigidity and motility, and RhoA-dependent endothelial contraction [81,82,83,84], which also involves c-ABL [85,86].

Increased cell stiffness implies a profound reorganization of the cellular cytoskeleton and/or the ECM [87,88,89]. As part of EndMT, we propose that the CMDV promotes intercellular junction disassembly and induces cell flattening, which would increase measurable cell stiffness. This likely depends on c-ABL and the Rho GTPase pathway [29]. The transient nature of the changes is likely due to the labile nature of the soluble factors [90]. 

EndMT increases cell stiffness as a consequence of the ECM composition and the shift in actin organization that swaps the polarity of the cells from apical-basolateral (epithelium/endothelium) to front-rear (migratory) [13,84,91]. Such a shift is also consistent with our in silico insights regarding the cellular effect of DEGs, which favor cell motility. It is expected that future validation of some of these targets will confirm these data and provide novel insights into the infectious mechanisms that govern cytoskeletal responses and cell stiffness. Also in agreement, we expect that the destabilization of adhesion junctions is an early sign of trans-differentiation. Cadherins and catenins are thus internalized into the cytoplasm [59]. 

The alteration to the dynamics of the junctions likely shifts the predominant mode of signaling from cell-to-cell adhesion, predominantly controlled by cadherins, to cell–matrix adhesion, which is mainly mediated by integrins. Integrin ligation triggers the RhoA/ROCK pathway, increasing actin contractility and the emergence of stress fibers [84,92,93,94]. Integrin ligation also activates focal adhesion kinase (FAK), allowing the formation of new focal adhesions, where talin (TLN) is key for the actin-integrin-ECM interaction and vinculin (VCL) stabilizes focal adhesions and modulates the mechanical forces that pass through them [95,96]. These changes shift the morphology of the cytoskeleton from endothelial to fibroblastic, increasing traction and consolidating the morphological shift. 

Accordingly, the morphological changes produced by the CMDV concur with decreased levels of zonula occludens 1 (ZO-1) and VE-cadherin, and increased levels of vimentin and N-cadherin, but only moderate differences in the reorganization of actin [29]. On the other hand, histological reports (post-mortem evidence) on DV-infected patients detected viral antigens in some types of endothelial cells (spleen and kidney), as well as the secretion of the Von Willebrand factor and Waibel–Palade bodies. These are increased in patients with severe dengue, explaining increased platelet adherence to endothelial cells in the liver, heart and other organs. Likewise, increased apoptosis has been reported, in patients with severe dengue who succumbed to the infection [26,97,98,99]. It is worth noting that the changes elicited by the CMDV are similar to those observed in HMEC-1 cells exposed to TGF-β at 48 h, which become mesenchymal but also display amoeboid protrusions consistent with increased RhoA activity [57].

The findings of this study provide valuable insights into the complex mechanisms underlying endothelial dysfunction in dengue disease, emphasizing the significant role of soluble factors beyond the virion in contributing to this condition. It also illustrates the potential as well as the limitations of in silico approaches to point towards new potential therapeutic targets. By investigating the effects of the conditioned media derived from DV-infected cells, this study reveals how these soluble factors can alter endothelial permeability and promote an EndMT-like process, highlighting potential targets for therapeutic intervention. This research advances our understanding of the pathophysiology of dengue and underscores the importance of targeting not only the virus but also the host’s response in mitigating severe disease outcomes. The early changes in endothelial function, such as increased permeability and cytoskeletal reorganization, point to potential intervention windows that could be exploited to prevent or reduce vascular complications in dengue.

## 4. Materials and Methods

### 4.1. Cell Lines and Viral Infections

Human microvascular endothelial cells (HMEC-1) (ATCC Cat# CRL-3243, RRID:CVCL_0307, Manassas, VA, USA) were maintained at 37 °C with 5% CO_2_ in RPMI supplemented with 10% FBS, 10 mM L-glutamine, 100 U/mL penicillin/100 mg/mL streptomycin (P/S), 10 ng/mL epidermal growth factor (hEGF), and 1 µg/mL hydrocortisone. Cells were used up to passage 10. *Aedes albopictus* clone C6/36 HT cells (ATCC Cat# CRL-1660, RRID:CVCL_Z230) were cultured at 34 °C with 5% CO_2_ in L-15 media supplemented with 10% FBS, 100 I.U./mL penicillin and 100 (μg/mL) streptomycin.

Dengue virus Serotype 2 (DENV-2, New Guinea) was amplified in C6/36 HT cells and titrated in BHK-21 (ATCC Cat# CCL-10, RRID:CVCL_1915) hamster kidney cells as described [100].

### 4.2. Conditioned Media

HMEC-1 cells were infected with the dengue virus serotype 2 (DENV-2) at a relative multiplicity of infection (MOI) of 5 for a duration of 2 h. Subsequently, the cells were rinsed with phosphate-buffered saline (PBS) and then cultured in RPMI supplemented with a 2% fetal bovine serum (FBS) for 48 h. The resultant cell culture supernatant was collected and transferred to Petri dishes, where the virus was subsequently inactivated using ultraviolet (UV) irradiation for 15 min. Finally, the inactivated supernatant was stored at −80 °C for further use [29].

### 4.3. Transendothelial Electrical Resistance (TEER)

A total of 4 × 10^4^ HMEC-1 cells were seeded per well in 0.4 µm-pore Transwell filters (Costar #3460, Arlington, VA, USA). The filter was previously treated with 4% porcine gelatin to enable cell adhesion. The cells were grown to confluency in RPMI1640 + 10% FBS (growth medium) at 37 °C under 5% CO_2_ atmosphere. After 24 h, the medium was changed to RPMI1640 + 0.1% FBS for synchronization and incubated for another 24 h. Then, the medium was changed to CMDV or growth medium supplemented with 5 ng/µL TGF-β1 (Sigma-Aldrich T7039, St. Louis, MO, USA) with restimulation every 24 h, or with the growth medium alone (control) for the times indicated in Figure 1.

TEER was measured in an EVOM2 and an EndOhm’s chamber (WPITM), at 48 h and 120 h after exposure to the CMDV. At 120 h, the cells exposed to CMDV were treated with 6.25 µM imatinib (STI571 from SelleckChem, Houston, TX, USA) and the TEER was measured at 48 and 120 h after treatment with the chemotherapeutic agent. To avoid cross-contamination, the Endohm chamber was sterilized with 70% ethanol and 96% isopropanol, followed by three washes with 1× PBS. Each assay included technical triplicates. The TEER was calculated according to the following equation [101]:

Relative TEER=(value (Ω)−average blank (Ω))  m_area,

where *m_area =* 1.12 cm^2^.

### 4.4. In-Cell Western Assay (ICW)

HMEC-1 cells were seeded at 1 × 10^4^ cells per well in the growth medium and incubated overnight at 37 °C under a 5% CO_2_ atmosphere. The cells were then treated with the CMDV for 48 and 120 h, or with 5 ng/mL TGF-β1 (re-stimulation every 24 h). After treatment, the cells were fixed with 3.8% paraformaldehyde and treated with 1× CytoBuster™ Protein Extraction Reagent (Sigma-Aldrich, St. Louis, MO, USA). Following fixation, the cells were incubated in a blocking solution (1× PBS with 5% FBS) at 37 °C, then stained using the following specific primary antibodies against: tubulin (Abbexa Cat# abx139709, RRID:AB_3665255, Cambridge, UK); SNAIL (Santa Cruz Biotechnology Cat# sc-271977, RRID:AB_10709902, Dallas, TX, USA); TWIST1 (Abbexa Cat# abx016017, RRID:AB_3665248); α-SMA (Abbexa Cat# abx015761, RRID:AB_3665254); TIE2 (Abbexa Cat# abx128494, RRID:AB_3665253). Primary antibodies were followed with goat anti-mouse labeled with Dylight800 (Thermo Fisher Scientific Cat# SA5-35521, RRID:AB_2556774, Waltham, MA, USA) or Dylight680 (Thermo Fisher Scientific Cat# 35518, RRID:AB_614942). The immunostaining intensity was measured in an Odyssey Infrared Imaging System (LiCOR, Lincoln, NE, USA) at 600 and 800 nm.

### 4.5. AFM Force Spectroscopy

AFM force spectroscopy experiments were performed on HMEC-1 cells in hydrated conditions. Briefly, 8.3 × 10^4^ HMEC-1 cells were seeded in 35 mm tissue culture dishes (Sigma-Aldrich, St. Louis, MO, USA). Cells were cultured overnight in RPMI1640 supplemented with 0.1% (FBS). Subsequently, the cells were exposed to various treatments: negative control was conditioned media from uninfected cells; CMDV for 48 h to detect early-intermediate changes [29]; or 120 h to enable complete trans-differentiation [14]. Following treatment, the cells were washed three times with 1× PBS and incubated with an incomplete RPMI1640 medium containing 25 mM HEPES for AFM measurements. AFM Force spectroscopy was performed using an Agilent 5500 (Keysight, Santa Rosa, CA, USA) and silicon dioxide particle probe (5 µm diameter, 0.5 µm Poisson radius, nominal spring constant κ ≈ 0.035 N m^−1^). The exact spring constant was measured using the N9469A cantilever spring constant calibration module incorporated into the AFM controller computer (NanoAndMore USA Inc., Watsonville, CA, USA). Cell samples were subjected to scanning in an area of 30 × 30 µm^2^ in three different zones. A force of 4 nN was applied during scanning, and the data was collected and processed using the Picoview v1.2 software. The cell’s Young modulus (E) was calculated by fitting the force curves using the classical Hertz model. Subsequent data analysis was performed using the AtomicJ v2.3.1 software (RRID:SCR_026023).

### 4.6. Bioinformatic Analysis

To investigate the transcriptome changes mediated by soluble factors induced by the DV virus infection, meta-analysis was performed using data from Human Umbilical Vein Endothelial Cells (HUVEC) exposed individually to different factors: ANG2, TNF-α, and VEGF. Relevant studies were retrieved from the Gene Expression Omnibus (GEO) (RRID:SCR_005012) database, and identified by the following GSE accessions: GSE77597, GSE2639, GSE9055, and GSE15464. Given the distinct nature of array-based data, preprocessing involved background correction, normalization, and the summarization of probe intensities. For the Affymetrix arrays, the robust multi-array average (RMA) normalization was applied using the oligo package in R software (v. 4.3.0, RRID:SCR_015729). The RMA performed background correction, quantile normalization, and log_2_ transformation to ensure comparability across arrays. For the Illumina arrays, the lumi package (RRID:SCR_012781) was employed for the variance-stabilizing transformation (VST) normalization. The lumiExpresso function was used to perform background correction and normalization. To address any potential batch effects or systematic variations across datasets, the ComBat function from the sva package (RRID:SCR_012836) was used.

Differential expression analysis was conducted using the Limma package in R. The normalized read counts from each dataset were analyzed to identify genes that were differentially expressed between HUVEC cells exposed to each soluble factor and control, untreated HUVEC cells. Statistical significance was determined based on adjusted *p*-values, controlling the false discovery rate (FDR). The Benjamini–Hochberg method [102] was used for multiple testing corrections, setting an FDR threshold of 0.05. Genes with an adjusted *p*-value below this threshold were considered differentially expressed. A fold-change cutoff was applied to identify genes with biologically meaningful changes in expression. Genes with an absolute fold change greater than a predefined threshold (1.5-fold) were considered differentially expressed.

The top 100 differentially expressed genes (DEGs) identified from the meta-analysis of both untreated and treated HUVEC cells were selected for further functional enrichment analysis. This analysis was performed using the Enrichr web tool (RRID:SCR_001575) [103], utilizing gene ontology (GO) annotation to identify the biological process enrichment, molecular functions, and cellular components associated with these DEGs.

### 4.7. Statistical Analysis

For the ICW and TEER assays, the data normality was assessed using the Shapiro–Wilk test. Depending on the distribution of the data, a one-way analysis of variance (ANOVA) was performed for the normally distributed data, while the Kruskal–Wallis test was used for the non-normally distributed data. For pairwise comparisons, t-tests were used for the normally distributed data, and the Mann–Whitney U test was applied for non-normally distributed data. Post-hoc analyses were conducted using Bonferroni or Dunnett tests as appropriate. For the AFM data, normality was similarly assessed with the Kolmogorov–Smirnov test. The statistical analyses included Bartlett’s test for the homogeneity of variances, followed by the Kruskal–Wallis test for non-parametric data. Pairwise comparisons of the AFM data were conducted using the Dunn–Bonferroni post-hoc test after the Kruskal–Wallis test. All statistical analyses and graphical representations were performed using GraphPad Prism v8.0 (RRID:SCR_002798) and RStudio v4.1.3, with packages including easypackages, base, dunn.test, stats, ggplot2, dplyr, lattice, DescTools, ggsci, and confidence.

## Figures and Tables

**Table 1:** Included studies in the meta-analysis of HUVEC cells exposed to DENV derived factors. Datasets were selected to simulate the soluble factors from the DV conditioned media.

GSE Accession	Treatment	Group (Treatment/Control)	Exposure Times	Number of Transcriptomes	Array Platform
GSE77597	Untreated	Control	0 h	2	Illumina HiSeq 2000 (Homo sapiens)
	ANG2 Activating Antibody	Treatment	4 h	2	Illumina HiSeq 2000 (Homo sapiens)
GSE2639	TNF	Treatment	5 h	4	Affymetrix Human Genome U133A Array
	Untreated	Control	0 h	4	Affymetrix Human Genome U133A Array
GSE9055	Untreated	Control	0 h	1	Affymetrix Human Genome U133 Plus 2.0 Array
	TNF	Treatment	From 1 to 8 h	21	Affymetrix Human Genome U133 Plus 2.0 Array
GSE15464	VEGF	Treatment	From 0.5 to 2.5 h	3	Affymetrix Human Genome U133 Plus 2.0 Array
	Untreated	Control	From 0.5 to 2.5 h	1	Affymetrix Human Genome U133 Plus 2.0 Array
GSE10778	VEGF	Treatment	From 0.5 to 6 h	4	Affymetrix Human Genome U133A Array

**Table 2:** Drug versus gene analysis.

Gene	Drug	Interaction Type	PMIDs
*CD69*	TOCILIZUMAB		27339827
*CDKN2B*	PALBOCICLIB		23898052
*CXCL10*	METHYLPREDNISOLONE		17220550
*CXCL8*	DACARBAZINE		
*HDAC9*	VORINOSTAT	inhibitor	19344175
*IFNGR1*	INTERFERON GAMMA-1B	Binder—agonist	17618444
*IGF1R*	PAZOPANIB		
*IL1R1*	ANAKINRA	Inhibitor—antagonist	17083033
*NT5C2*	MERCAPTOPURINE		15990089
*PDGFD*	SUNITINIB	inhibitor	
*PIK3CD*	IDELALISIB	inhibitor	
*PMAIP1*	BORTEZOMIB		16024631
*PTK2*	PAZOPANIB		
*SMAD2*	BLEOMYCIN		17274978
*SOD2*	PACLITAXEL		25495407
*TNF*	THALIDOMIDE	inhibitor	8755512
*TNFSF13B*	BELIMUMAB	Antibody—inhibitor—modulator	
*VCAM1*	DEXAMETHASONE		7694584
*GAS6*	DOCETAXEL		27153245
*BCL2L11*	IMATINIB		24223824

Possible pharmacological targets obtained from the DGIdb using the DEGs resulting from the exposure of HUVEC cells to DV-soluble factors.

## Data Availability

Original data are available from the authors upon reasonable request.

## Supplementary Materials

The supporting information can be downloaded at: https://www.mdpi.com/article/10.3390/ijms26052139/s1.

## References

[1] Madewell Z.J. (2020). Arboviruses and Their Vectors. South. Med. J..

[2] Messina J.P., Brady O.J., Golding N., Kraemer M.U.G., Wint G.R.W., Ray S.E., Pigott D.M., Shearer F.M., Johnson K., Earl L. (2019). The Current and Future Global Distribution and Population at Risk of Dengue. Nat. Microbiol..

[3] Soneja S., Tsarouchi G., Lumbroso D., Tung D.K. (2021). A Review of Dengue’s Historical and Future Health Risk from a Changing Climate. Curr. Environ. Health Rep..

[4] Deng S.Q., Yang X., Wei Y., Chen J.T., Wang X.J., Peng H.J. (2020). A Review on Dengue Vaccine Development. Vaccines.

[5] Mishra R., Lata S., Ali A., Banerjea A.C. (2019). Dengue Haemorrhagic Fever: A Job Done via Exosomes?. Emerg. Microbes Infect..

[6] Paranavitane S.A., Gomes L., Kamaladasa A., Adikari T.N., Wickramasinghe N., Jeewandara C., Shyamali N.L.A., Ogg G.S., Malavige G.N. (2014). Dengue NS1 Antigen as a Marker of Severe Clinical Disease. BMC Infect. Dis..

[7] Bandara S.M.R., Herath H.M.M.T.B. (2020). Corticosteroid Actions on Dengue Immune Pathology; A Review Article. Clin. Epidemiol. Glob. Health.

[8] Malavige G.N., Ogg G.S. (2017). Pathogenesis of Vascular Leak in Dengue Virus Infection. Immunology.

[9] Halstead S. (2019). Recent Advances in Understanding Dengue. F1000Research.

[10] Bhatt P., Sabeena S.P., Varma M., Arunkumar G. (2021). Current Understanding of the Pathogenesis of Dengue Virus Infection. Curr. Microbiol..

[11] Guzman M.G., Harris E. (2015). Dengue. Lancet.

[12] Srikiatkhachorn A., Kelley J.F. (2014). Endothelial Cells in Dengue Hemorrhagic Fever. Antivir. Res..

[13] Piera-Velazquez S., Jimenez S.A. (2019). Endothelial to Mesenchymal Transition: Role in Physiology and in the Pathogenesis of Human Diseases. Physiol. Rev..

[14] Pinto M.T., Covas D.T., Kashima S., Rodrigues C.O. (2016). Endothelial Mesenchymal Transition: Comparative Analysis of Different Induction Methods. Biol. Proced. Online.

[15] Zhang B., Niu W., Dong H.Y., Liu M.L., Luo Y., Li Z.C. (2018). Hypoxia Induces Endothelial-Mesenchymal Transition in Pulmonary Vascular Remodeling. Int. J. Mol. Med..

[16] Echeverría C., Montorfano I., Tapia P., Riedel C., Cabello-Verrugio C., Simon F. (2014). Endotoxin-Induced Endothelial Fibrosis Is Dependent on Expression of Transforming Growth Factors Β1 and Β2. Infect. Immun..

[17] Huang X., Pan L., Pu H., Wang Y., Zhang X., Li C., Yang Z. (2013). Loss of Caveolin-1 Promotes Endothelial-Mesenchymal Transition during Sepsis: A Membrane Proteomic Study. Int. J. Mol. Med..

[18] Saito A. (2013). EMT and EndMT: Regulated in Similar Ways?. J. Biochem..

[19] Naipauer J., Mesri E.A. (2023). The Kaposi’s Sarcoma Progenitor Enigma: KSHV-Induced MEndT–EndMT Axis. Trends Mol. Med..

[20] Ciszewski W.M., Woźniak L.A., Sobierajska K. (2024). Diverse Roles of SARS-CoV-2 Spike and Nucleocapsid Proteins in EndMT Stimulation through the TGF-β-MRTF Axis Inhibited by Aspirin. Cell Commun. Signal..

[21] Álvarez-Díaz D.A., Gutiérrez-díaz A.A., Orozco-garcía E., Puerta-gonzález A., Bermúdez-santana C.I., Gallego-gómez J.C. (2019). Dengue Virus Potentially Promotes Migratory Responses on Endothelial Cells by Enhancing Pro-Migratory Soluble Factors and MiRNAs. Virus Res..

[22] Roa Linares V.C., Gallego Gómez J.C. (2019). La Pérdida de Función de La Quinasa Dependiente de Ciclina 5 (CDK5) Altera El Citoesqueleto y Reduce La Infección in Vitro Por El Virus Del Dengue 2. Acta Biol. Colomb..

[23] Suttitheptumrong A., Mahutchariyakul T., Rawarak N., Reamtong O., Boonnak K., Pattanakitsakul S.N. (2021). Altered Moesin and Actin Cytoskeleton Protein Rearrangements Affect Transendothelial Permeability in Human Endothelial Cells upon Dengue Virus Infection and Tnf-α Treatment. Viruses.

[24] Dalrymple N.A., MacKow E.R. (2012). Roles for Endothelial Cells in Dengue Virus Infection. Adv. Virol..

[25] Rathi K.R., Arora M.M., Sahai K., Tripathi S., Singh S.P., Raman D.K., Anand K.B. (2013). Autopsy Findings in Fatal Dengue Haemorrhagic Fever—06 Cases. Med. J. Armed Forces India.

[26] Póvoa T.F., Alves A.M.B., Oliveira C.A.B., Nuovo G.J., Chagas V.L.A., Paes M.V. (2014). The Pathology of Severe Dengue in Multiple Organs of Human Fatal Cases: Histopathology, Ultrastructure and Virus Replication. PLoS ONE.

[27] Xu X., Zhang Y., Wang X., Li S., Tang L. (2021). Substrate Stiffness Drives Epithelial to Mesenchymal Transition and Proliferation through the Neat1-Wnt/β-Catenin Pathway in Liver Cancer. Int. J. Mol. Sci..

[28] Mahdi A., Jiao T., Tratsiakovich Y., Wernly B., Yang J., Östenson C.G., Danser A.H.J., Pernow J., Zhou Z. (2022). Therapeutic Potential of Sunitinib in Ameliorating Endothelial Dysfunction in Type 2 Diabetic Rats. Pharmacology.

[29] Escudero-Flórez M., Torres-Hoyos D., Miranda-Brand Y., Boudreau R.L., Gallego-Gómez J.C., Vicente-Manzanares M. (2023). Dengue Virus Infection Alters Inter-Endothelial Junctions and Promotes Endothelial–Mesenchymal-Transition-like Changes in Human Microvascular Endothelial Cells. Viruses.

[30] Siavashi V., Nassiri S.M., Rahbarghazi R., Mohseni Z., Sharifi A.M. (2019). Distinct Tie2 Tyrosine Phosphorylation Sites Dictate Phenotypic Switching in Endothelial Progenitor Cells. J. Cell. Physiol..

[31] Vitali R., Mancini C., Cesi V., Tanno B., Mancuso M., Bossi G., Zhang Y., Martinez R.V., Calabretta B., Dominici C. (2008). Slug (SNAI2) down-Regulation by Interference Facilitates Apoptosis and Inhibits Invasive Growth in Neuroblastoma Preclinical Models. Clin. Cancer Res..

[32] Anupriya M.G., Singh S., Hulyalkar N.V., Sreekumar E. (2018). Sphingolipid Signaling Modulates Trans-Endothelial Cell Permeability in Dengue Virus Infected HMEC-1 Cells. Prostaglandins Other Lipid Mediat..

[33] Velandia-Romero M.L., Calderón-Peláez M.A., Castellanos J.E. (2016). In Vitro Infection with Dengue Virus Induces Changes in the Structure and Function of the Mouse Brain Endothelium. PLoS ONE.

[34] Velandia-Romero M.L., Caldern-Pelaez M.A., Balbas-Tepedino A., Alejandro Marquez-Ortiz R., Madroñero L.J., Prieto A.B., Castellanos J.E. (2020). Extracellular Vesicles of U937 Macrophage Cell Line Infected with DENV-2 Induce Activation in Endothelial Cells EA.Hy926. PLoS ONE.

[35] Wang J.L., Zhang J.L., Chen W., Xu X.F., Gao N., Fan D.Y., An J. (2010). Roles of Small GTPase RAc1 in the Regulation of Actin Cytoskeleton during Dengue Virus Infection. PLoS Negl. Trop. Dis..

[36] Singh S., Anupriya M.G., Sreekumar E. (2017). Comparative Whole Genome Analysis of Dengue Virus Serotype-2 Strains Differing in Trans-Endothelial Cell Leakage Induction in Vitro. Infect. Genet. Evol..

[37] Dewi B.E., Takasaki T., Kurane I. (2004). In Vitro Assessment of Human Endothelial Cell Permeability: Effects of Inflammatory Cytokines and Dengue Virus Infection. J. Virol. Methods.

[38] Dewi B.E., Takasaki T., Kurane I. (2008). Peripheral Blood Mononuclear Cells Increase the Permeability of Dengue Virus-Infected Endothelial Cells in Association with Downregulation of Vascular Endothelial Cadherin. J. Gen. Virol..

[39] Luplerdlop N., Missé D., Bray D., Deleuze V., Gonzalez J.P., Leardkamolkarn V., Yssel H., Veas F. (2006). Dengue-Virus-Infected Dendritic Cells Trigger Vascular Leakage through Metalloproteinase Overproduction. EMBO Rep..

[40] Bok K., Castagnaro N., Borsa A., Nates S., Espul C., Fay O., Fabri A., Grinstein S., Miceli I., Matson D.O. (2001). Plasma Concentrations of SVCAM-1 and Severity of Dengue Infections. J. Med. Virol..

[41] Kelley J.F., Kaufusi P., Nerurkar V. (2012). Dengue Hemorrhagic Fever-Associated Immunomediators Induced via Maturation of Dengue Virus Nonstructural 4B Protein in Monocytes Modulate Endothelial Cell Adhesion Molecules and Human Microvascular Endothelial Cells Permeability. Virology.

[42] Warke R.V., Xhaja K., Martin K.J., Fournier M.V., Shaw S.K., Brizuela N., de Bosch N., Lapointe D., Ennis F.A., Rothman A.L. (2004). Dengue Virus Induces Novel Changes in Gene Expression of Human Umbilical Vein Endothelial Cells. J. Virol..

[43] Fiestas Solórzano V.E., de Lima R.C., de Azeredo E.L. (2022). The Role of Growth Factors in the Pathogenesis of Dengue: A Scoping Review. Pathogens.

[44] Fiestas Solórzano V.E., da Costa Faria N.R., dos Santos C.F., Corrêa G., Cipitelli M.d.C., Dornelas Ribeiro M., de Souza L.J., Damasco P.V., da Cunha R.V., dos Santos F.B. (2021). Different Profiles of Cytokines, Chemokines and Coagulation Mediators Associated with Severity in Brazilian Patients Infected with Dengue Virus. Viruses.

[45] Jeewandara C., Gomes L., Wickramasinghe N., Gutowska-Owsiak D., Waithe D., Paranavitane S.A., Shyamali N.L.A., Ogg G.S., Malavige G.N. (2015). Platelet Activating Factor Contributes to Vascular Leak in Acute Dengue Infection. PLoS Negl. Trop. Dis..

[46] Puerta-Guardo H., Glasner D.R., Harris E. (2016). Dengue Virus NS1 Disrupts the Endothelial Glycocalyx, Leading to Hyperpermeability. PLoS Pathog..

[47] Glasner D.R., Puerta-Guardo H., Beatty P., Harris E. (2018). The Good, the Bad, and the Shocking: The Multiple Roles of Dengue Virus Nonstructural Protein 1 in Protection and Pathogenesis. Annu. Rev. Virol..

[48] Barbachano-Guerrero A., Endy T.P., King C.A. (2020). Dengue Virus Non-Structural Protein 1 Activates the P38 MAPK Pathway to Decrease Barrier Integrity in Primary Human Endothelial Cells. J. Gen. Virol..

[49] Chanthick C., Suttitheptumrong A., Rawarak N., Pattanakitsakul S.N. (2018). Transcytosis Involvement in Transport System and Endothelial Permeability of Vascular Leakage during Dengue Virus Infection. Viruses.

[50] Wang C., Puerta-Guardo H., Biering S., Glasner D.R., Tran E.B., Patana M., Gomberg T.A., Malvar C., Lo N.T.N., Espinosa D. (2019). Endocytosis of Flavivirus NS1 Is Required for NS1-Mediated Endothelial Hyperpermeability and Is Abolished by a Single N-Glycosylation Site Mutation. PLoS Pathog..

[51] Dalrymple N.A., Mackow E. (2011). Productive Dengue Virus Infection of Human Endothelial Cells Is Directed by Heparan Sulfate-Containing Proteoglycan Receptors. J. Virol..

[52] Modhiran N., Watterson D., Muller D., Panetta A.K., Sester D., Liu L., Hume D., Stacey K., Young P. (2015). Dengue Virus NS1 Protein Activates Cells via Toll-like Receptor 4 and Disrupts Endothelial Cell Monolayer Integrity. Sci. Transl. Med..

[53] Modhiran N., Watterson D., Blumenthal A., Baxter A., Young P., Stacey K. (2017). Dengue Virus NS1 Protein Activates Immune Cells via TLR4 but Not TLR2 or TLR6. Immunol. Cell Biol..

[54] Sabbineni H., Verma A., Somanath P.R. (2018). Isoform-Specific Effects of Transforming Growth Factor β on Endothelial-to-Mesenchymal Transition. J. Cell. Physiol..

[55] Dong W., Kong M., Zhu Y., Shao Y., Wu D., Lu J., Guo J., Xu Y. (2020). Activation of TWIST Transcription by Chromatin Remodeling Protein BRG1 Contributes to Liver Fibrosis in Mice. Front. Cell Dev. Biol..

[56] Ladak S., McQueen L., Tomkova K., Adebayo A., Suleiman S., George S., Murphy G., Zakkar M. (2023). Dexamethasone Modulate TWIST Mediated EndMT Changes in Venous EC under Acute Shear Stress. Implications for Vein Grafts Disease. medRxiv.

[57] Kryczka J., Przygodzka P., Bogusz H., Boncela J. (2017). HMEC-1 Adopt the Mixed Amoeboid-Mesenchymal Migration Type during EndMT. Eur. J. Cell Biol..

[58] Stasiak M., Gawryś K., Popielarski M., Bednarek R., Studzian M., Sitkiewicz E., Szemraj J., Świątkowska M. (2017). Differential Quantitative Proteomics of Human Microvascular Endothelial Cells 1 by ITRAQ Reveals Palladin to Be a New Biomarker During TGF-Β1 Induced Endothelial Mesenchymal Transition. J. Proteom. Bioinform..

[59] Islam S., Boström K.I., Di Carlo D., Simmons C.A., Tintut Y., Yao Y., Hsu J.J. (2021). The Mechanobiology of Endothelial-to-Mesenchymal Transition in Cardiovascular Disease. Front. Physiol..

[60] Ciszewski W.M., Sobierajska K., Wawro M.E., Klopocka W., Chefczyńska N., Muzyczuk A., Siekacz K., Wujkowska A., Niewiarowska J. (2017). The ILK-MMP9-MRTF Axis Is Crucial for EndMT Differentiation of Endothelial Cells in a Tumor Microenvironment. Biochim. Biophys. Acta (BBA)-Mol. Cell Res..

[61] Mimouni M., Lajoix A.D., Desmetz C. (2024). Experimental Models to Study Endothelial to Mesenchymal Transition in Myocardial Fibrosis and Cardiovascular Diseases. Int. J. Mol. Sci..

[62] Van Meeteren L.A., Ten Dijke P. (2012). Regulation of Endothelial Cell Plasticity by TGF-β. Cell Tissue Res..

[63] Yoshimatsu Y., Watabe T. (2022). Emerging Roles of Inflammation-Mediated Endothelial–Mesenchymal Transition in Health and Disease. Inflamm. Regen..

[64] Kovacic J.C., Kishta F., Xu Y., Baker A.H. (2024). Endothelial to Mesenchymal Transition: At the Axis. Cardiovasc. Res..

[65] Chislock E.M., Ring C., Pendergast A.M. (2013). Abl Kinases Are Required for Vascular Function, Tie2 Expression, and Angiopoietin-1-Mediated Survival. Proc. Natl. Acad. Sci. USA.

[66] Jia W., Wang Z., Gao C., Wu J., Wu Q. (2021). Trajectory Modeling of Endothelial-to-Mesenchymal Transition Reveals Galectin-3 as a Mediator in Pulmonary Fibrosis. Cell Death Dis..

[67] Di Benedetto P., Ruscitti P., Berardicurti O., Vomero M., Navarini L., Dolo V., Cipriani P., Giacomelli R. (2021). Endothelial-to-Mesenchymal Transition in Systemic Sclerosis. Clin. Exp. Immunol..

[68] Aono Y., Nishioka Y., Inayama M., Ugai M., Kishi J., Uehara H., Izumi K., Sone S. (2005). Imatinib as a Novel Antifibrotic Agent in Bleomycin-Induced Pulmonary Fibrosis in Mice. Am. J. Respir. Crit. Care Med..

[69] Akhmetshina A., Venalis P., Dees C., Busch N., Zwerina J., Schett G., Distler O., Distler J.H.W. (2009). Treatment with Imatinib Prevents Fibrosis in Different Preclinical Models of Systemic Sclerosis and Induces Regression of Established Fibrosis. Arthritis Rheum..

[70] Touret F., Baronti C., Goethals O., Van Loock M., de Lamballerie X., Querat G. (2019). Phylogenetically Based Establishment of a Dengue Virus Panel, Representing All Available Genotypes, as a Tool in Dengue Drug Discovery. Antivir. Res..

[71] Vedagiri D., Gupta D., Mishra A., Krishna G., Bhaskar M., Sah V., Basu A. (2021). Retinoic Acid-Inducible Gene I-Like Receptors Activate Snail To Limit RNA Viral Infections. J. Virol..

[72] Alfaro-García J.P., Granados-Alzate M.C., Vicente-Manzanares M., Gallego-Gómez J.C. (2021). An Integrated View of Virus-Triggered Cellular Plasticity Using Boolean Networks. Cells.

[73] Frakolaki E., Kaimou P., Moraiti M., Kalliampakou K.I., Karampetsou K., Dotsika E., Liakos P., Vassilacopoulou D., Mavromara P., Bartenschlager R. (2018). The Role of Tissue Oxygen Tension in Dengue Virus Replication. Cells.

[74] Hincapie V., Gallego-gómez J.C. (2020). Transición Epitelio-Mesénquima Inducida Por Virus. Acta Biol. Colomb..

[75] Clark M.J., Miduturu C., Schmidt A.G., Jang J., Chu H., Gray N.S., Yang P.L., Clark M.J., Miduturu C., Schmidt A.G. (2016). GNF-2 Inhibits Dengue Virus by Targeting Abl Kinases and the Viral E Protein Article GNF-2 Inhibits Dengue Virus by Targeting Abl Kinases and the Viral E Protein. Cell Chem. Biol..

[76] Sharma S., Sicinski P. (2020). A Kinase of Many Talents: Non-Neuronal Functions of CDK5 in Development and Disease. Open Biol..

[77] Chislock E.M., Pendergast A.M. (2013). Abl Family Kinases Regulate Endothelial Barrier Function In Vitro and in Mice. PLoS ONE.

[78] Zhou Z., Wu Y.F., Wang X., Han Y.Z. (2017). The C-Abl Inhibitor in Parkinson Disease. Neurol. Sci..

[79] Wang X., Bleher R., Wang L., Garcia J.G.N., Dudek S.M., Shekhawat G.S., Dravid V.P. (2017). Imatinib Alters Agonists-Mediated Cytoskeletal Biomechanics in Lung Endothelium. Sci. Rep..

[80] Talavera D., Castillo A.M., Dominguez M.C., Escobar Gutierrez A., Meza I. (2004). IL8 Release, Tight Junction and Cytoskeleton Dynamic Reorganization Conducive to Permeability Increase Are Induced by Dengue Virus Infection of Microvascular Endothelial Monolayers. J. Gen. Virol..

[81] Lutter S., Xie S., Tatin F., Makinen T. (2012). Smooth Muscle–Endothelial Cell Communication Activates Reelin Signaling and Regulates Lymphatic Vessel Formation. J. Cell Biol..

[82] Dugina V.B., Shagieva G.S., Shakhov A.S. (2021). The Cytoplasmic Actins in the Regulation of Endothelial Cell Function. Int. J. Mol. Sci..

[83] Molla R., Shimizu A., Komeno M., Rahman N.I.A., Ern J., Soh C., Kim L., Nguyen C., Khan M.R., Tesega W.W. (2022). Vascular Smooth Muscle RhoA Counteracts Abdominal Aortic Aneurysm Formation by Modulating MAP4K4 Activity. Commun. Biol..

[84] Ciszewski W.M., Wawro M.E., Sacewicz-hofman I., Sobierajska K. (2021). Cytoskeleton Reorganization in Endmt—The Role in Cancer and Fibrotic Diseases. Int. J. Mol. Sci..

[85] Hawez A., Ding Z., Taha D., Madhi R. (2021). C-Abl Kinase Regulates Neutrophil Extracellular Trap Formation and Lung Injury in Abdominal Sepsis. Nature.

[86] Amado J., Anne A., Stalborch M.D.V., Valent E.T., Nawaz K., Bezu J.V., Abl-related A., Crk-like C. (2021). Depletion of Arg/Abl2 Improves Endothelial Cell Adhesion and Prevents Vascular Leak during Inflammation Acute Respiratory Distress Syndrome. Angiogenesis.

[87] Deville S.S., Cordes N. (2019). The Extracellular, Cellular, and Nuclear Stiffness, a Trinity in the Cancer Resistome—A Review. Front. Oncol..

[88] DeWane G., Salvi A.M., DeMali K.A. (2021). Fueling the Cytoskeleton-Links between Cell Metabolism and Actin Remodeling. J. Cell Sci..

[89] Danielsson F., Peterson M.K., Araújo H.C., Lautenschläger F., Gad A.K.B. (2018). Vimentin Diversity in Health and Disease. Cells.

[90] Takaoka Y., Uchinomiya S., Kobayashi D., Endo M., Hayashi T., Fukuyama Y., Hayasaka H., Miyasaka M., Ueda T., Shimada I. (2018). Endogenous Membrane Receptor Labeling by Reactive Cytokines and Growth Factors to Chase Their Dynamics in Live Cells. Chem.

[91] Zhong A., Mirzaei Z., Simmons C.A. (2018). The Roles of Matrix Stiffness and SS-Catenin Signaling in Endothelial-to-Mesenchymal Transition of Aortic Valve Endothelial Cells. Cardiovasc. Eng. Technol..

[92] Barry A.K., Wang N., Leckband D.E. (2015). Local VE-Cadherin Mechanotransduction Triggers Long-Ranged Remodeling of Endothelial Monolayers. J. Cell Sci..

[93] Kechagia J.Z., Ivaska J., Roca-Cusachs P. (2019). Integrins as Biomechanical Sensors of the Microenvironment. Nat. Rev. Mol. Cell Biol..

[94] Wang W., Wang Z., Tian D., Zeng X., Liu Y., Fu Q., Liang A., Zhang Y., Gao Q., Cheng J. (2018). Integrin Β3 Mediates the Endothelial-to-Mesenchymal Transition via the Notch Pathway. Cell. Physiol. Biochem..

[95] Martino F., Perestrelo A.R., Vinarský V., Pagliari S., Forte G. (2018). Cellular Mechanotransduction: From Tension to Function. Front. Physiol..

[96] Ma X., Geng Z., Wang S., Yu Z., Liu T., Guan S., Du S., Zhu C. (2023). The Driving Mechanism and Targeting Value of Mimicry between Vascular Endothelial Cells and Tumor Cells in Tumor Progression. Biomed. Pharmacother..

[97] Rivera J., Neira M., Parra E., Méndez J., Sarmiento L., Caldas M.L. (2014). Detección de Antígenos Del Virus Del Dengue En Tejidos Post Mórtem. Biomedica.

[98] Rivera J.A., Rengifo A.C., Parra E.A., Castellanos J.E., Caldas M.L. (2020). Histopatología Ilustrada de Casos Fatales de Dengue En Colombia. Biomedica.

[99] Vásquez Ochoa M., García Cordero J., Gutiérrez Castañeda B., Santos Argumedo L., Villegas Sepúlveda N., Cedillo Barrón L. (2009). A Clinical Isolate of Dengue Virus and Its Proteins Induce Apoptosis in HMEC-1 Cells: A Possible Implication in Pathogenesis. Arch. Virol..

[100] Martinez-Gutierrez M., Correa-Londoño L.A., Castellanos J.E., Gallego-Gómez J.C., Osorio J.E. (2014). Lovastatin Delays Infection and Increases Survival Rates in AG129 Mice Infected with Dengue Virus Serotype 2. PLoS ONE.

[101] Srinivasan B., Kolli A.R., Esch M.B., Abaci H.E., Shuler M.L., Hickman J.J. (2015). TEER Measurement Techniques for In Vitro Barrier Model Systems. J. Lab. Autom..

[102] Wilcox R. (2012). One-Way and Higher Designs for Independent Groups. Introduction to Robust Estimation and Hypothesis Testing.

[103] Subramanian A., Tamayo P., Mootha V.K., Mukherjee S., Ebert B.L., Gillette M.A., Paulovich A., Pomeroy S.L., Golub T.R., Lander E.S. (2005). Gene Set Enrichment Analysis: A Knowledge-Based Approach for Interpreting Genome-Wide Expression Profiles. Proc. Natl. Acad. Sci. USA.

