# Supplementary material for: Characterization of the Temporal Dynamics of the Endothelial–Mesenchymal-like Transition Induced by Soluble Factors from Dengue Virus Infection in Microvascular Endothelial Cells
Source: Int J Mol Sci. 2025 Feb 27;26(5):2139. doi: 10.3390/ijms26052139 (PMC11900998; doi:10.3390/ijms26052139)
Supplement: Supplementary file 1 [file ijms-26-02139-s001.zip › manuscript.v11_MV Figure S1.pdf]

International Journal of
Molecular Sciences

MDPI

Article

Characterization of the Temporal Dynamics of the Endothelial–Mesenchymal-like Transition Induced by Soluble Factors from Dengue Virus Infection in Microvascular Endothelial Cells

Jenny Paola Alfaro-García [1], Carlos Alberto Orozco-Castaño [2], Julián Andrés Sánchez-Rendón [3], Herley Fernando Casanova-Yépes [3], Miguel Vicente-Manzanares [4,*,†] and Juan Carlos Gallego-Gómez [1,*,†]

Supplementary Data

Plaque Unit Formation assay

To verify the observed effect was produced by the CMDV and not by an active virion, Plaque Unit Formation assay was performed with the protocol by Escudero et al., 2023, using Vero Cells (ATCC Cat# CCL-81, RRID:CVCL_0059). The cells were seeded at $5.5x10^4$ cells per well in DMEM with 5% FBS overnight to allow cellular adhesion.

To compare the results obtained, other multiwell with VERO cells (at the same density) were previously infected with Dengue Virus as described in Escudero et al.,2023, with the only difference being that infection was performed in 75 cm^2 flasks in the former study. On the sixth day, the cells were fixed with a solution containing 3.5% PFA and 0.2% formaldehyde–crystal violet and plaque-forming-cells were imaged in a EVOS5000 microscope (Thermo).

Supplementary Figure 1 shows the CMDV viral titer plate (a) and there is not a visible marked effect on the fixed cells that can be attributed to an active virus. To verify the absence of plaque-forming-cells, the plate was observed under microscopy and the highest concentration was registered (CMDV (10^{-1})) (c).

The results were compared with the negative control (b) and the monolayer is not completely like the healthy cells (c-), but, when the CMDV results are compared with an active Dengue Virus infection (10-1) (d) CMDV does not produce plaque-forming-cells, as seen in active infection conditions (some of the plaque-forming-cells found are highlighted in a red circle and red arrows). This confirmed that CMDV does not contain active virions and the alterations are produced by the soluble factors contained in the media.

 https://doi.org/10.3390ijms26052139

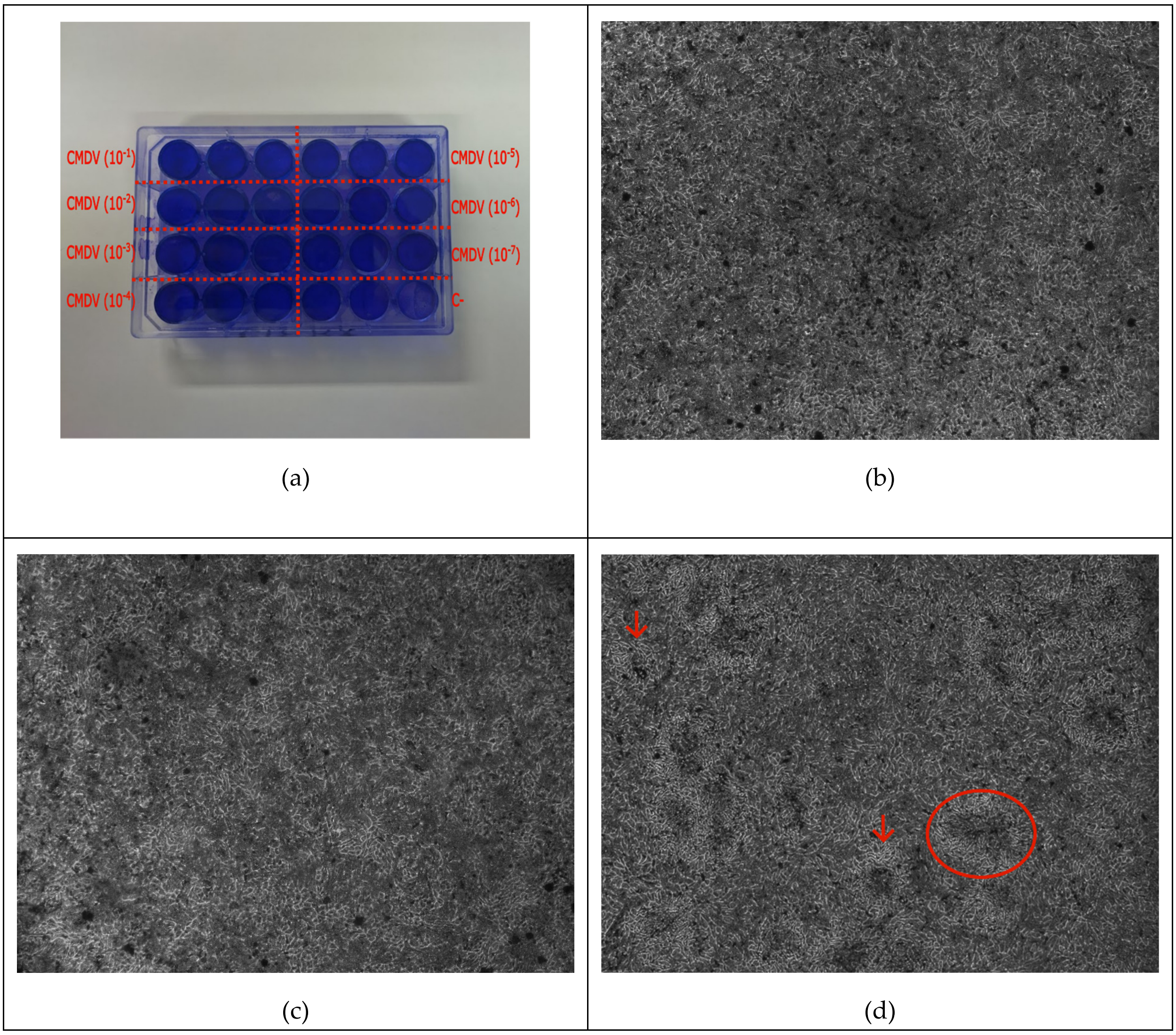

Supplementary Figure S1 Plaque Unit Formation assay using CMDV. (a) Titer plate with all the dilutions performed and not visible viral effects. (b) Negative control. Cells exposed to conditioned media from healthy cells (c) CMDV (10-^{1}) (d) Active Dengue Virus Infection (10-^{1}) and plaque-forming-cells in the monolayer.
